# Deep Batch Integration and Denoise of Single‐Cell RNA‐Seq Data

**DOI:** 10.1002/advs.202308934

**Published:** 2024-05-22

**Authors:** Lu Qin, Guangya Zhang, Shaoqiang Zhang, Yong Chen

**Affiliations:** ^1^ College of Computer and Information Engineering Tianjin Normal University Tianjin 300387 China; ^2^ Department of Biological and Biomedical Sciences Rowan University NJ 08028 USA

**Keywords:** batch effect, cell typing, data integration, deep learning, scRNA‐seq

## Abstract

Numerous single‐cell transcriptomic datasets from identical tissues or cell lines are generated from different laboratories or single‐cell RNA sequencing (scRNA‐seq) protocols. The denoising of these datasets to eliminate batch effects is crucial for data integration, ensuring accurate interpretation and comprehensive analysis of biological questions. Although many scRNA‐seq data integration methods exist, most are inefficient and/or not conducive to downstream analysis. Here, DeepBID, a novel deep learning‐based method for batch effect correction, non‐linear dimensionality reduction, embedding, and cell clustering concurrently, is introduced. DeepBID utilizes a negative binomial‐based autoencoder with dual Kullback–Leibler divergence loss functions, aligning cell points from different batches within a consistent low‐dimensional latent space and progressively mitigating batch effects through iterative clustering. Extensive validation on multiple‐batch scRNA‐seq datasets demonstrates that DeepBID surpasses existing tools in removing batch effects and achieving superior clustering accuracy. When integrating multiple scRNA‐seq datasets from patients with Alzheimer's disease, DeepBID significantly improves cell clustering, effectively annotating unidentified cells, and detecting cell‐specific differentially expressed genes.

## Graphical abstract

This work presents DeepBID, a novel deep learning‐based solution for batch integration and denoising of scRNA‐seq data. DeepBID not only corrects batch effects but also enables non‐linear dimensionality reduction, embedding, and clustering simultaneously, resulting in superior performance compared to existing methods. DeepBID showcases remarkable enhancements in cell clustering, cell annotation, and identification of differentially expressed genes in Alzheimer's disease research.

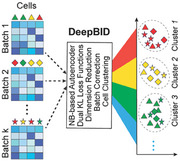

## 1 Introduction

Single‐cell sequencing has enabled the study of cell heterogeneity and molecular mechanisms in individual cells. In recent years, due to the widespread application of single‐cell RNA sequencing (scRNA‐seq), a vast amount of scRNA‐seq datasets of the same cell lines or tissues have been generated under different experimental conditions or laboratories, especially in several important cell atlas projects.^[^ ^1^, ^2^, ^3^, ^4^ ^]^ However, comprehensive analysis of these scRNA‐seq datasets is challenging and limits the discovery of biological insights due to the inevitable nonlinear batch effects.^[^ ^5^ ^]^ Batch effects in scRNA‐seq datasets refer to systematic differences in gene expression measurements that arise from technical variations introduced during sample processing, library preparation, and sequencing. These variations can occur between different experimental batches, such as samples processed on different days or using different protocols, and can confound the biological signal in the data. Batch effects can significantly impact downstream analysis, leading to spurious associations, false positives, and decreased reproducibility. Consequently, developing efficient methods to denoise data, eliminate batch effects, and integrate multiple datasets has emerged as a priority in the comprehensive analysis of scRNA‐seq data.^[^ ^6^ ^]^

Many methods for removing batch effects have been developed, and their performance has been compared using common benchmarking data.^[^ ^5^, ^7^ ^]^ Integration methods generally operate under the assumption that shared cell types exist across different batches, or at least, some cells of the same type are present across batches. Three technical strategies are commonly used for batch effect removal: *k*‐nearest neighbors (KNN), mutual nearest neighbors (MNN),^[^ ^8^ ^]^ and canonical correlation analysis (CCA).^[^ ^9^ ^]^ For example, Seurat4^[^ ^10^ ^]^ employs diagonalized CCA with L_2_ normalization to jointly reduce the dimensionality of datasets from two different batches, and then it searches for MNNs in the shared low‐dimensional subspace across datasets. BBKNN^[^ ^11^ ^]^ applies principal component analysis (PCA) for dimensionality reduction and identifies KNNs for each cell in each batch independently. Subsequently, it utilizes the UMAP^[^ ^12^ ^]^ algorithm to transform the neighbor information into the connectivity of the neighbor sets across batches. DeepMNN^[^ ^13^ ^]^ searches for MNN pairs across different batches in a PCA subspace and a batch correction network with a stack of two residual blocks is constructed based on these MNN pairs. Scanorama^[^ ^14^ ^]^ applies randomized singular value decomposition (SVD) to cell‐by‐gene expression matrices to learn a low‐dimensional embedding of each cell. Then, it merges cells from different batches with similar embeddings using an approximate MNN strategy paired with a panorama stitching strategy. scDML^[^ ^15^ ^]^ first employs a graph‐based clustering algorithm to cluster cells within each batch and then uses KNN and MNN information both within and between batches to evaluate the similarity between cell clusters hierarchically. RPCI (Reference Principal Component Integration)^[^ ^16^ ^]^ independently decomposes the cell‐by‐gene expression matrix of each batch dataset using SVD based on a global reference gene eigenvector and projects all cells into a global reference RPCI space. Harmony^[^ ^17^ ^]^ utilizes PCA to achieve a low‐dimensional embedding of all batches and then employs soft *k*‐means clustering to group cells of each batch into clusters. After clustering, it repeatedly learns cluster‐specific linear correction factors and adjusts cell assignments until they are stabilized. LIGER^[^ ^18^ ^]^ first employs integrative non‐negative matrix factorization (iNMF) to decompose the data into shared factors and dataset‐specific factors, through which it identifies cell types that are common across datasets and those unique to specific conditions.

In addition to using dimensionality reduction strategies such as CCA, PCA, and SVD, several deep‐learning‐based methods, including BERMUDA,^[^ ^19^ ^]^ scVI,^[^ ^20^ ^]^ iMAP,^[^ ^21^ ^]^ and DESC,^[^ ^22^ ^]^ perform data integration by mapping the cell profiles from each batch to a low‐dimensional latent space within a neural network. BERMUDA^[^ ^19^ ^]^ first clusters cells from each batch and then removes batch effects using an autoencoder with a transfer loss that is determined by estimating the difference in distributions between pairs of cell clusters. scVI^[^ ^20^ ^]^ operates as a deep generative model, utilizing deep neural networks and stochastic optimization to obtain a probabilistic representation of scRNA‐seq data with minimized batch effects. iMAP^[^ ^21^ ^]^ builds a deep model with an encoder designed to extract low‐dimensional, batch‐agnostic representations of each cell profile and two generators for profile reconstruction. Subsequently, a generative adversarial network (GAN) structure is trained on extended MNN pairs from two batches to further eliminate batch effects. DESC^[^ ^22^ ^]^ uses a stacked autoencoder to learn a low‐dimensional representation of cell profiles. The resulting encoder is then added to a neural network to cluster cells iteratively with a Kullback–Leibler (KL) divergence loss.

While these computational methods have shown potential in addressing batch effects in scRNA‐seq data, there remain several technical limitations hindering their performance. First, MNN‐based techniques are primarily devised to integrate only two batches simultaneously. As a result, these methods lack a consistent global reference space for seamlessly integrating multiple datasets. Second, existing deep learning‐based methods often overlook the underlying data distribution. Recent statistical analyses of scRNA‐seq data, especially from mainstream commercial platforms like 10X genomics, indicate that the gene expression profiles among cells typically follow a negative binomial (NB) distribution.^[^ ^23^, ^24^, ^25^, ^26^ ^]^ Implementing an autoencoder that fits the NB distribution has been shown to enhance data imputation and the accuracy of cell clustering.^[^ ^27^ ^]^ For example, BERMUDA^[^ ^19^ ^]^ and SAUCIE^[^ ^28^ ^]^ employ NB distributions, whereas DESC^[^ ^22^ ^]^ and scDeepCluster^[^ ^29^ ^]^ utilize zero‐inflated negative binomial (ZINB) distributions to model gene expression counts in clustering analysis of scRNA‐seq data. However, many other methods do not explicitly incorporate the distributions of gene expression counts. Third, only a few algorithms, such as DESC,^[^ ^22^ ^]^ offer simultaneous data integration and cell clustering, which streamlines subsequent data analysis. In contrast, methods like Harmony,^[^ ^17^ ^]^ BERMUDA,^[^ ^19^ ^]^ LIGER,^[^ ^18^ ^]^ and Seurat4^[^ ^10^ ^]^ treat data integration and clustering as distinct steps in their pipelines. For example, BERMUDA^[^ ^19^ ^]^ first clusters cells within each batch before merging clusters from different batches. Meanwhile, most of the current methods optimize between either dimensionality reduction and integration (e.g., RPCI, scVI, and iMAP), integration and clustering (e.g., Harmony), or dimensionality reduction and clustering (e.g., DESC). Integrating the removal of batch effects with downstream analysis such as cell clustering can be beneficial in improving performance, facilitating benchmarking, increasing robustness, and offering benefits in computational scalability and efficiency. Hence, there is a significant need for a method that can coordinate optimization across dimensionality reduction, integration, and clustering.^[^ ^30^ ^]^ To address this, we introduce DeepBID (Deep Batch Integration and Denoise), a method that facilitates cell clustering in a low‐dimensional latent space while progressively mitigating batch effects during cell profile reconstruction. Nonlinear dimension reduction, batch effect removal, and clustering are optimized by employing fuzzy *k*‐means on an NB‐based autoencoder, which incorporates two KL divergence losses into the adaptive loss function of fuzzy *k*‐means. Extensive validation tests on multi‐batch datasets and real applications to multiple datasets from patient samples with Alzheimer's Disease (AD) demonstrate that DeepBID is highly effective in denoising batch effects, and cell clustering, and consistently outperforms other well‐known tools. When applied to multiple scRNA‐seq datasets from AD patients, DeepBID significantly improved clustering results, effectively annotating unidentified cells and detecting cell‐specific differentially expressed genes (DEGs).

## 2 Results

### 2.1 Overview of DeepBID

**Figure 1:**
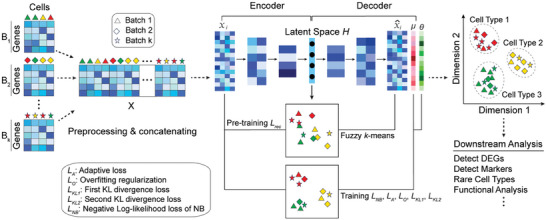
The workflow of the DeepBID method. DeepBID processes scRNA‐seq datasets from multiple batches as inputs. It employs an NB‐based autoencoder for pretraining to optimize the latent space, enabling iterative integration and clustering. The loss function is composed of multiple subfunctions that cater to nonlinear dimension reduction, batch effect removal, and clustering, while also preventing overfitting. The outputs of DeepBID include an integrated gene expression matrix and clustered cell types, ready for various downstream analyses. The various shapes (such as triangles, diamonds, and stars) represent different batches, while the distinct colors denote different cell types.

DeepBID is designed to efficiently denoise batch effects and integrate multiple scRNA‐seq datasets by leveraging advanced deep learning strategies (**Figure** **1**). Its primary optimization goal is to achieve a close overlap of cells from different batches in the integrated space while maintaining a clear separation between different cell types. It employs an autoencoder to map all cell profiles *X* from different batches to a common, non‐linear, low‐dimensional latent feature space *H*. The encoder component of the autoencoder is responsible for reducing the dimensionality of input data from multiple batches, while the decoder reconstructs the data. To denoise the input data, an NB distribution is integrated by appending two independent fully connected layers to the last reconstruction layer, aiming to estimate its mean and dispersion parameters. To cluster closely related cells effectively, the objective function of fuzzy *k*‐means with weighted adaptive loss is embedded within the latent feature space *H* of the autoencoder.^[^ ^31^ ^]^ The solution of fuzzy clustering is a soft assignment matrix of cells *P*  = (*p*_*jk*_)_*N* × *K*_, where *p*_*jk*_ is the probability that cell *j* belongs to the *k*‐th cluster. To preserve and amplify the association between similar cells, a specific KL divergence between soft assignment and an auxiliary distribution for each cell is used as a loss function, a technique employed by some clustering algorithms such as scziDesk.^[^ ^32^ ^]^ Moreover, to ensure that cells in the same cluster originate from different batches, a second KL divergence loss is introduced to measure the disorder of cells from various batches within each cluster. Finally, to counteract overfitting in DeepBID, a regularization loss is incorporated into the total loss function.

The DeepBID software is designed for user‐friendly operation within a PyTorch environment and offers flexibility by accommodating a variety of user‐defined parameters. Its input can consist of multiple scRNA‐seq datasets from different batches, whether from the same experiment, the same tissue samples, or even different labs. The software outputs an integrated gene expression matrix and cell type annotation, which can be differently used for downstream analysis (right side, Figure 1). Furthermore, DeepBID features an option to use CPU+GPU hybrid computing for processing extensive datasets. The inputs and outputs of DeepBID can be directly integrated with popular scRNA‐seq analysis pipelines like SCANPY.^[^ ^33^ ^]^

### 2.2 Hyperparameter Selections of DeepBID for Diverse Datasets

**Figure 2:**
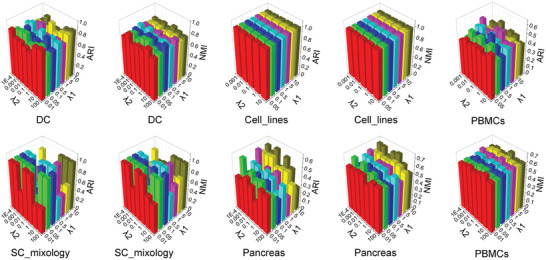
Clustering results of ARI and NMI scores on the five datasets for different *λ*_1_ and *λ*_2_ with fixed *κ*_1_ =  0.01 and *κ*_2_ =  100.

DeepBID possesses four hyperparameters in its final objective function: *λ*_1_, *λ*_2_, *κ*_1_, and *κ*_2_ (refer to Equation 11 in Experimental Section). We initially assessed the performance of DeepBID with various values for *λ*_1_ and *λ*_2_ while keeping *κ*_1_ =  0.01 and *κ*_2_ =  100 fixed. Adjusted Rand index (ARI)^[^ ^34^ ^]^ and normalized mutual information (NMI)^[^ ^35^ ^]^ scores were used to evaluate the quality of clustering results. ARI measures the similarity between two sets of cluster assignments while NMI measures the mutual information between the true labels and the clustering results, normalizing to account for differences in cluster sizes. High ARI and NMI scores indicate strong agreement between the true labels and the clustering results. Here, NMI and ARI scores were computed for five datasets: “DC”, “Cell_lines”, “SC_mixology”, “Pancreas”, and “PBMCs”, which were sequenced either by a single sequencing platform or generated through multiple platforms (see Table S1, Supporting Information). All five datasets possess labels for batches and cell types and have been utilized as benchmarks in various integration or clustering tools.^[^ ^14^, ^17^, ^21^, ^22^ ^]^ As indicated in **Figure** **2**, *λ*_1_ =  5 and *λ*_2_ =  0.01 yield excellent outcomes across these datasets. Notably, for the two large‐scale datasets “Pancreas” and “PBMCs” (with total cell numbers exceeding 10,000), the optimal settings are *λ*_1_ =  1 and *λ*_2_ =  0.0001. Consequently, we recommend setting *λ*_2_ to be 0.01 for small‐scale datasets and 0.0001 for large‐scale datasets to prevent model overfitting.

After determining the values of *λ*_1_ and *λ*_2_, we proceeded to select suitable values for the hyperparameters *κ*_1_ and *κ*_2_, which represent the two KL divergence losses. Fixing *λ*_1_ and *λ*_2_ values (*λ*_1_ =  5 and *λ*_2_ =  0.01 for the datasets “DC”, “Cell_lines” and “Sc_mixology”, and *λ*_1_ =  1 and *λ*_2_ =  0.0001 for “Pancreas” and “PBMCs”), we executed DeepBID using seven distinct *κ*_1_ values (i.e., 0.001, 0.01, 0.1, 0, 1, 10, or 100). When the 20 epochs were used, the ARI and NMI scores peaked at *κ*_1_ =  0.01 for four of the five datasets (Figure S1, Supporting Information). However, the “PBMCs” dataset displayed more consistent results under *κ*_1_ =  0.01 compared to *κ*_1_ =  1. Therefore, we uniformly adopted *κ*_1_ =  0.01 for all datasets. Notably, clustering results with *κ*_1_ =  0 significantly underperformed those with *κ*_1_ =  0.01, underscoring the importance of proper parameter configurations for the primary KL divergence loss in enhancing clustering accuracy.

The purpose of the secondary KL divergence is to amplify the integration effect. We experimented with a range of *κ*_2_ values from 0.001 to 1,000 while maintaining *λ*_1_, *λ*_2_ and *κ*_1_ =  0.01. After 20 epochs, the clustering outcomes of the integrated data at *κ*_2_ =  100 were more resilient compared to other settings for most of the datasets (Figure S2, Supporting Information). Although the “Cell_lines” dataset remained largely unchanged across different *κ*_2_ configurations, the results at *κ*_2_ =  100 outpaced those at *κ*_2_ =  0. This indicated that the secondary KL divergence loss played a crucial role in achieving precise integration and clustering. This was especially evident in large‐scale datasets like “Pancreas” and “PBMCs”, where the benefits of *κ*_2_ =  100 were particularly pronounced. Therefore, we set hyperparameters *κ*_1_ =  0.01,   *κ*_2_ =  100 in the practical applications of DeepBID. Furthermore, we observed that DeepBID could achieve stable NMI scores after 20 epochs (Figures S1 and S2, Supporting Information). Thus, DeepBID sets the default number of epochs to 20 to balance performance and runtimes.

### 2.3 DeepBID Performs Better Than the Other Five Methods in Batch Integration

**Figure 3:**
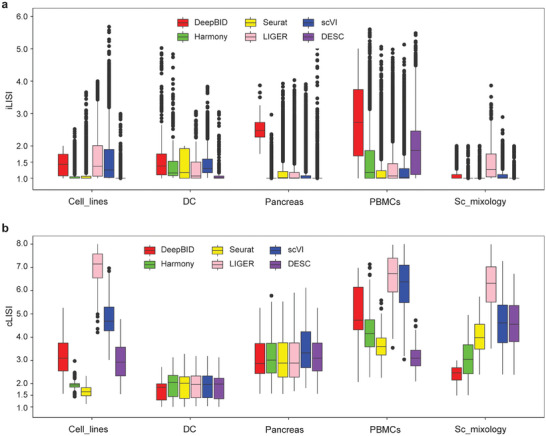
Validation results of data integration of six methods across five datasets. a) Boxplots of the iLISI scores for six methods on five datasets. The outline dots represent the extreme values in each dataset, which are 1.5 times of the interquartile range. b) Boxplots of the cLISI scores for six methods on five datasets.

**Figure 4:**
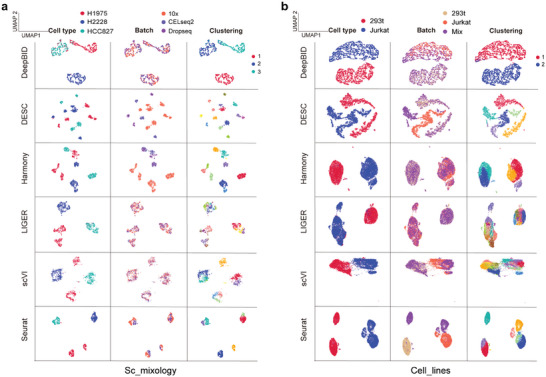
UMAP plots of six methods on the “Sc_mixology” and “Cell_lines” datasets. a) UMAP plots on the “Sc_mixology” dataset that has three cell types. The first column illustrates the original cell type labels by method, the second column the batch labels, and the third column the clustering outcomes after batch effect removal. b) UMAP plots on the “Cell_lines” dataset with two cell types. The figures in the first column show the labels of two cell types for each method. Columns are organized similarly to (a), showing original cell type labels, batch labels, and clustering outcomes.

We computed LISI (Local Inverse Simpson Index)^[^ ^17^ ^]^ to evaluate the effect of data integration. LISI has two specific metrics, iLISI (i.e., integration LISI) and cLISI (i.e., cell‐type LISI), which measure the number of batches and the number of cell types within a local neighborhood, respectively. We applied six different methods to each dataset to integrate various batches. We first constructed box plots and determined the median iLISI score for every method on each dataset (**Figure** **3**a; Table S2, Supporting Information). It is important to note that a higher iLISI score indicates a greater mixing degree of different batches. Results showed that DeepBID boasted the highest median iLISI scores for four datasets: “Cell_lines”, “DC”, “Pancreas”, and “PBMCs”. The only exception was the “Sc_mixology” dataset, where DeepBID's median iLISI score was surpassed by LIGER's. However, the clustering performance of DeepBID was markedly superior to that of LIGER. Specifically, we visualized the UMAP plots for both raw and integrated cells on the “Sc_mixology” dataset. The visual representation revealed that DeepBID accurately segregated cells into three clusters consistent with cell types (**Figure** **4**a). In contrast, LIGER partitioned them into nine clusters, even though it recorded the highest median iLISI scores. Moreover, the remaining four methods segmented the “Sc_mixology” cells into more than six clusters. Taken together, these results suggested that DeepBID integration aligned more closely with cell types than LIGER.

We further calculated the cLISI score, which indicates the proximity of cells of the same type, with lower scores indicating closer similarity. As shown in Figure 3b and Table S3 (Supporting Information), DeepBID achieved the lowest median cLISI scores on three datasets: “DC”, “Sc_mixology”, and “Pancreas”. Although the median cLISI score of DeepBID was higher than those of Harmony, Seurat, and DESC for the “Cell_lines” and “PBMCs” datasets, its clustering results were superior to the other six methods for these datasets. This could be explained by differences in cluster compaction across methods. For illustration, the UMAP plots for the “Cell_lines” dataset, as produced by the six methods, are showcased in Figure 4b. The cell clusters produced by DeepBID appear to be more scattered relative to Harmony, Seurat, and DESC. DeepBID distinctly partitioned the dataset into two groups, closely reflecting the two cell types: “293T” and “Jurkat”. Contrastingly, DESC, Harmony, and Seurat identified more than two domains, thereby introducing false positives. Therefore, solely relying exclusively on LISI scores, without precise clustering, compromises the accurate assessment of batch integration efficacy. DeepBID provided the most effective integration among the six methods for the “Sc_mixology” and “Cell_lines” datasets, even if its LISI scores were suboptimal.

### 2.4 DeepBID Performs Better Than the Other Five Methods in Cell Clustering

**Figure 5:**
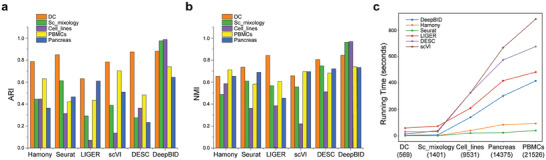
Performances comparison of six methods across five datasets. Cell clustering by six methods after batch removal across five datasets. a) ARI scores. b) NMI scores. c) Running time. The cell numbers for each dataset are provided in parentheses, and detailed runtimes are available in Table S5 (Supporting Information).

To investigate whether data integration could enhance cell clustering, we applied DeepBID and five other methods to five benchmark datasets (detailed in Experimental Section). We calculated the ARI and NMI scores for all clustering results and discovered that DeepBID consistently outperformed the other methods (**Figure** **5**; Table S4, Supporting Information). Specifically, DeepBID demonstrated better clustering performance on the “Sc_mixology” and “Cell_lines” datasets. The ARI score of DeepBID on “Sc_mixology” was 0.99146, which was 1.6 times higher than the second‐best score (0.61212) achieved by the Seurat method (Figure 5a). On the “Cell_lines” dataset, the ARI score of DeepBID is 0.99821, which was more than double the second‐best score (0.44557) generated by Harmony. Similar patterns were observed with the NMI scores (Figure 5b). On the “Sc_mixology” dataset, DeepBID achieved an NMI score of 0.98498, which was 1.3 times the second‐best score (0.74793) generated by DESC. On the “Cell_lines” dataset, the NMI score of DeepBID is 0.97854, 1.6 times the second‐best score (0.58549) by the Harmony method. The high performance of DeepBID was also observed on other datasets. For the “DC” dataset, both DeepBID and DESC performed better than the rest, while on the “PBMCs” dataset, DeepBID, scVI, and Harmony emerged as the top three for clustering efficiency. On the “Pancreas” dataset, DeepBID and LIGER achieved the highest ARI scores compared to the other four methods.

As deep learning‐based methods typically demand more runtime for model training and prediction compared to conventional statistical approaches, assessing runtimes across datasets of varying sizes is imperative. This becomes especially crucial as scRNA‐seq datasets may expand to millions of cells in large research projects.^[^ ^36^, ^37^ ^]^ To address this, we recorded the runtime performance for six methods on the five datasets with increasing cell numbers: DC (569 cells), Sc_mixology (1,401 cells), Cell_lines (9,531 cells), Pancreas (14,375 cells), and PBMCs (21,526 cells). The runtime measurement captured the core processing of each tool but excluded preprocessing time. The results, summarized in Table S5 (Supporting Information) and plotted in Figure 5c, highlighted DeepBID's relatively low runtimes across all datasets. For instance, it processed the DC dataset of 569 cells in only 4.45 seconds (s) and the PBMC dataset of 21,526 cells in 416.5 s. We observed a nearly linear increase in runtime with increasing cell numbers. Although DeepBID is slower than two non‐deep learning‐based methods, Harmony and Seurat, it outperforms the other three deep learning‐based methods in terms of speed.

Beyond these compared criteria, we further plotted and manually examined the cell clusters for the five datasets. First, we observed that although the cells identified by DeepBID seem more dispersed compared to other methods, DeepBID effectively preserved the true cell type clusters (see “Sc_mixology” and “Cell_lines” in Figure 4). Other methods tended to produce artificial clusters, suggesting potential artifact introduction during batch effect removal. Thus, these methods may introduce pseudo‐cell types into real data analysis. Second, for datasets with many cell types, such as “DC” (Figure S3, Supporting Information), “Pancreas” (Figure S4, Supporting Information), and “PBMCs” (Figure S5, Supporting Information), methods like LIGER and scVI often lead to merged cell clusters, indicating a failure to adequately separate cell types after batch effect adjustment. Overall, these results demonstrate that DeepBID consistently outperformed existing methods in integration and deep clustering across these benchmark datasets.

### 2.5 DeepBID Enhances Clustering Performance and Downstream DEG Analysis in Integrating Multiple AD scRNA‐seq Datasets

To provide a comprehensive demonstration of DeepBID's efficacy and its downstream analytical advantages, we applied DeepBID to three scRNA‐seq datasets obtained from three AD patients. These datasets, denoted as AD1‐2, AD3‐4, and AD5‐6, were sourced from post‐mortem entorhinal cortex tissue and characterized by the presence of 8 annotated cell types.^[^ ^38^ ^]^ As a demonstration, we sequentially conducted cell clustering and DEG detection on these datasets, both with and without the application of DeepBID batch correction. Subsequently, we compared their results to assess their improvements.

**Figure 6:**
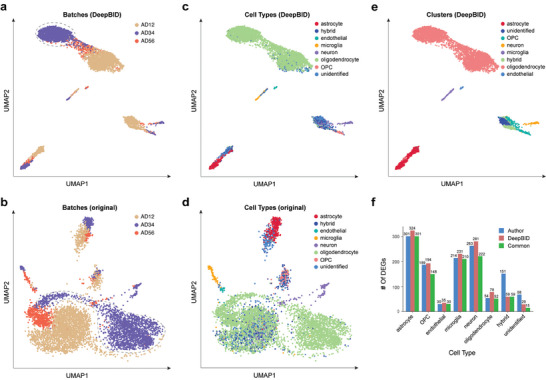
DeepBID enhances clustering performance and downstream analysis for multiple scRNA‐seq datasets from AD patients. a) UMAP plot of DeepBID's latent space marked for the three datasets. The dotted line highlights the largest cell group in the AD3‐4 dataset. b) UMAP plot of the merged original three datasets without batch correction. c) UMAP plot of DeepBID's latent space marked for eight cell types with original annotations. OPC (Oligodendrocyte progenitor cell). d) UMAP plot of the merged original three datasets without batch correction. Eight cell types are marked with original annotations. e) New clustering based on DeepBID's latent space. Eight cell types were manually annotated in this research. f) Comparison of the DEGs of eight cell types. The DEGs were calculated by using the authors’ clustering result and DeepBID analysis respectively. Commonly detected DEGs are shown in green.

We first evaluated whether DeepBID could enhance clustering performance. The UMAP plot of DeepBID's latent space illustrates that cells from different datasets aggregate more closely than those plotted from the original datasets (**Figure** **6**a,b). For instance, the largest cell group (annotated as oligodendrocytes) in the AD3‐4 dataset disperses widely based on original data (Figure 6b) but converges into a densely packed group after DeepBID batch correction (marked in a dotted circle in Figure 6a). Furthermore, these oligodendrocytes exhibit close aggregations with those in the AD1‐2 and AD5‐6 datasets (Figure 6a,c). In contrast, oligodendrocytes primarily overlap with neurons and unidentified cells when using original data (Figure 6d). Integrating these three datasets, DeepBID effectively clustered the eight‐cell types (Figure 6e), achieving a high ARI score of 0.8239, significantly higher than the score of 0.6912 calculated without batch correction, and the score of 0.7458 obtained with the second‐ranked method, LIGER.

We then investigated whether the enhanced clustering results by DeepBID could improve the detection of cell‐specific DEGs. For each of the eight cell types, we predicted the DEGs based on the original authors' labels and the clustering results obtained using DeepBID. Interestingly, the results revealed that in six cell types, the number of detected DEGs increased compared to the original analysis (Figure 6f; Table S7, Supporting Information). These six cell types (microglia, astrocytes, neurons, oligodendrocyte progenitor cells, oligodendrocytes, and endothelial cells) are all well‐annotated cell types in previous studies.^[^ ^38^ ^]^ Additionally, we observed a decrease in DEGs for two cell types (i.e., hybrid and unidentified), which are not well‐defined cell types. Furthermore, the changes in DEGs are associated with the methodological differences in clustering results. For example, DeepBID increased the number of astrocytes to 548, compared to the 472 astrocytes originally annotated in the author's analysis. Those astrocytes are closely co‐localized with some unidentified cells in the original annotation (red dots and light blue dots in Figure 6c), but our clustering results suggest that all the cells in the cluster would be astrocytes (red dots in Figure 6e). Meanwhile, the number of cells clustered as unidentified decreased to 278 (blue dots in Figure 6e), compared to the original clustered number of 614 (light blue dots in Figure 6c). Taken together, the increased number of DEGs in well‐annotated cell types and the decreased number of DEGs in unidentified cell types indicate that DeepBID can indeed improve the cell clusters, consequently enhancing DEG analysis.

## 3 Discussion

Adjusting batch effects in multiple scRNA‐seq datasets is challenging but essential for downstream computational analysis. Deep learning methods have gained popularity for their potential to model complex nonlinear relationships within data, making them particularly suitable for correcting batch effects in scRNA‐seq data. In this direction, our method, DeepBID, effectively facilitates cell clustering in a low‐dimensional latent space and progressively mitigates batch effects during cell profile reconstruction. When evaluated across five datasets, featuring varying numbers of batches, DeepBID demonstrates superior performance over five other well‐established methods. This enhanced accuracy is primarily attributed to two sophisticated techniques implemented in DeepBID: the NB‐based autoencoder for data imputation according to the NB distribution, and the simultaneous optimization of batch integration and clustering of data.

Integrating multiple scRNA‐seq datasets is not purely a data‐driven, but rather a problem‐driven approach. We would like to highlight that efforts to remove batch effects, combined with downstream analysis such as clustering, can indeed be beneficial for several reasons. First, incorporating batch correction into analysis workflows preserves signals relevant to the biological questions at hand. Compared with traditional batch correction methods, which may remove or alter biological signals while correcting for batch effects, an integrated approach could minimize this risk by directly optimizing for the preservation of relevant biological information during batch correction. Second, batch correction can be made more robust by directly observing its effects on downstream tasks, thus facilitating result interpretation. Finally, merging batch correction with downstream analysis can be more computationally efficient, as it avoids the need to store and manage separate intermediate representations of the data. Thus, this integrative strategy has the great potential to enhance the accuracy and biological relevance of diverse single‐cell data analysis. It is noteworthy that different downstream tasks might be differently affected by batch effects, depending on the specific biological context. For example, batch effects can introduce systematic biases in gene expression profiles across experiments, affecting the estimation of pseudotime and the accuracy of trajectory inference.^[^ ^39^ ^]^ Meanwhile, cells from different batches may be incorrectly positioned along the trajectory, leading to distorted or inaccurate representations of developmental trajectories. Thus, it is reasonable to carefully analyze these potential factors and design proper loss functions accordingly.

While DeepBID performs well on the datasets used for validation, there are several potential directions for further development. First, it is important to continue testing on a wide range of datasets to optimize hyper‐parameters to various types of batch effects across different tissues, cell types, and sequencing platforms. Second, with the emergence of multi‐single‐cell omics, a possible direction could be adapting DeepBID to integrate scRNA‐seq data with other types of single‐cell data, such as scATAC‐seq,^[^ ^40^ ^]^ scHi‐C,^[^ ^41^, ^42^, ^43^ ^]^ and methylation data.^[^ ^44^ ^]^ Third, DeepBID can be complemented by other strategies to effectively detect rare cell populations. For instance, a recent deep learning‐based method called DeepScena is specifically tailored for hierarchical detection of rare cell populations and has demonstrated superior performance compared to other methods.^[^ ^27^ ^]^ Therefore, an updated approach to detect rare cell types could involve utilizing DeepBID to integrate multiple batches and subsequently applying DeepScena for hierarchical detection. These iterative refinements and enhanced robustness will further bolster the applicability of DeepBID in addressing evolving scRNA‐seq datasets and analysis requirements.

## 4 Experimental Section

DeepBID projected the data of all batches onto a non‐linear low‐dimensional latent space *H* and simultaneously learned a soft assignment matrix *P* of all cells in the latent space. It minimized a total loss function consisting of the NB‐based loss, an adaptive loss, a regularization loss, and two KL divergence losses (Figure 1).

### Data Preprocessing

Before deploying DeepBID, the Python package SCANPY^[^ ^33^ ^]^ (version 1.9.2) was employed to preprocess the raw expression count matrices of all batches of a scRNA‐seq dataset to select highly variable genes (HVGs) and simply merge batch data. Supposedly, a scRNA‐seq dataset contained *t* batches *B*_1_,*B*_2_,⋅⋅⋅, *B_t_* with corresponding count matrices *M*_1_,*M*_2_,…, *M*_*t*_. For each batch matrix *M*_*b*_, the ‘*scanpy.pp.filter_genes*’ function was applied to filter out genes with non‐zero counts in fewer than three cells. Next, the ‘*scanpy.pp.normalize_total*’ function was used to normalize the counts per cell by the total counts across all genes, with a size factor of 10^4^. This was followed by a log transformation of the normalized matrix by using the ‘*scanpy.pp.log1p*’ function. It then employed the *‘scanpy.pp.highly_variable_genes*’ function to select the top 1000 HVGs. Lastly, the ‘*scanpy.AnnData.concatenate*’ function was used to concatenate the normalized matrices to retain only the union of the selected HVGs from all batches. This process, comprising the normalization and HVG selection procedures in SCANPY, accomplished preliminary data integration. Subsequently, DeepBID integrated the preprocessed concatenated matrix *X* of all batches through soft clustering based on an NB‐based autoencoder with two KL divergence losses and additional loss functions.

### NB‐Based Denoising Autoencoder

As the NB distribution was widely used in characterizing gene expression in scRNA‐seq data,^[^ ^23^, ^24^, ^25^ ^]^ an NB‐based denoising autoencoder was applied. This autoencoder mapped the input cell profiles to an embedded latent space *H* to perform both batch integration and cell clustering.

For the preprocessed matrix $X=\left(X_{1},X_{2},\text{…},X_{N}\right)\;\in R^{f\times N},$ where *N* represents the total number of cells and *X_j_* = (*X*_*1* *j*_,*X*_*2* *j*_,⋅⋅⋅, *X*_*fj*_)^*T*^  is an *f*‐dimensional vector representing the profiles of *f* selected feature genes of the *j*‐th cell, it was assumed that each element *X*_*ij*_ of *X* conformed to an NB distribution parameterized with mean *µ*_*ij*_ and dispersion *θ*_*ij*_. That is,

(1) $$P_{\mathrm{NB}}\;\left(\left[X_{\mathrm{ij}}\right]|\mu ,\theta \right)=\frac{\Gamma \left(\left[X_{\mathrm{ij}}\right]+\theta_{\mathrm{ij}}\right)}{\left[X_{\mathrm{ij}}\right]!\Gamma \left(\theta_{\mathrm{ij}}\right)}\;{\left(\frac{\theta_{\mathrm{ij}}}{\theta_{\mathrm{ij}}+\mu_{\mathrm{ij}}}\right)}^{\theta_{\mathrm{ij}}}{\left(\frac{\mu_{\mathrm{ij}}}{\theta_{\mathrm{ij}}+\mu_{\mathrm{ij}}}\right)}^{\left[X_{\mathrm{ij}}\right]}$$

where [*X*_*ij*_] is the integer obtained by rounding the preprocessed expression value *X*_*ij*_. To predict the putative true transcriptional profiles, in addition to the reconstruction output layer $\hat{X}$, two output layers are appended to estimate the two parameter sets of the NB distribution, specifically the mean set *µ* and the dispersion set *θ*, as shown in Figure 1.

The encoder function was defined as *H*  = *f*_*w*_ (*X*) and the decoder function $\hat{X}=g_{w^{\prime }}\;\left(H\right)$, where *W* and *W* *′* are the learned weights of the encoder and decoder functions, respectively. To introduce non‐linearity and learn complex relationships in the data, both the encoder and decoder functions consisted of fully connected neural networks with “tanh” activation. The "tanh” function was characterized by a smooth, S‐shaped curve that mapped input values to output values in the range from −1 to 1. If *D* represents the last hidden layer of the decoder, two independent fully connected output layers were added to *D* to estimate the mean set *µ * = exp (*W*_*µ*_ *D*)  and the dispersion set *θ*  = exp (*W*_*θ*_ *D*) , respectively, where *W*_*µ*_ and *W*_*θ*_ are the learned weights from the last hidden layer of the decoder to the two output layers, respectively. The exponential function was used as the activation function for the mean and dispersion parameters due to their non‐negativity. The loss function of the NB‐based autoencoder was formulated as the negative log‐likelihood of NB distribution:

(2) $$L_{\mathrm{NB}}\;\left(\mu ,\theta \right)=-\log\left(P_{\mathrm{NB}}\left(X|\mu ,\theta \right)\right)=-\sum_{j\;=\;1}^{N}\sum_{i\;=\;1}^{f}\log\left(P_{\mathrm{NB}}\left(X_{\mathrm{ij}}|\mu_{\mathrm{ij}},\theta_{\mathrm{ij}}\right)\right)$$

### Deep Fuzzy Clustering with Adaptive Loss Function and Regularization

Supposedly, all input cell profiles $\left(X_{1},X_{2},\text{…},X_{N}\right)\in R^{f\times N}$ were projected into a *d*‐dimensional latent space, represented as $H=\left(h_{1},h_{2},\text{…},h_{N}\right)\;\in R^{d\times N}$. Here, $h_{j}\in R^{d}$ is a low‐dimensional representation of $X_{j}\in R^{f}$. A fuzzy *k*‐means clustering with an adaptive loss *L*_*A*_ in the latent space and a regularization term *L_O_* to prevent overfitting in each layer were performed.^[^ ^31^ ^]^ Let there be *K* clusters with centers *C*  =  (*c*_*1*_,…, *c*_*k*_,…, *c*_*K*_) in the latent space. To force each cell point *h*_*j*_ to move closer to its nearest cluster center *c*_*k*_, *L*_*A*_ is defined as

(3) $$L_{A}=\sum_{j=1}^{N}\sum_{k=1}^{K}\left(d_{\mathrm{jk}}p_{\mathrm{jk}}∥h_{j}-c_{k}{∥}_{2}^{2}\right)$$

where $d_{\mathrm{jk}}=\frac{∥h_{j}-c_{k}{∥}_{2}+2}{{\left(∥h_{j}-c_{k}{∥}_{2}+1\right)}^{2}}$ and ‖ · ‖_2_ represents $l_{2}$‐norm. *L*_*O*_ is defined as

(4) $$L_{O}=\sum_{m=1}^{M}\left(∥W^{\left(m\right)}{∥}_{F}^{2}+∥b^{\left(m\right)}{∥}_{2}^{2}\right)$$

where *W* ^(*m*)^ and *b* ^(*m*)^ are the weight matrix and bias of the *m*‐th layer of the autoencoder, *M* is the total number of layers in the autoencoder, and ${∥\cdot ∥}_{F}^{2}$ represents the squared Frobenius norm of a matrix. Thus, the objective function of the fuzzy *k*‐means with the NB‐based autoencoder can be defined as

(5) $$\min_{W,b,P,C}L_{\mathrm{NB}}+\lambda_{1}L_{A}+\lambda_{2}L_{O}$$

(6) $$s.t.\;\sum_{k\;=\;0}^{K}\;p_{\mathrm{jk}}=1,\;0\leq p_{\mathrm{jk}}\leq 1,\;j=1,2,\text{…},N$$

### Integration by Deep Clustering with Two KL Divergence Losses

The fuzzy *k*‐means clustering previously described does not account for the pairwise distances and movements of similar cells. Ideally, clustering should group similar cells from the same batch into the same cluster. To facilitate this, a KL divergence loss function, as the one used in scziDesk,^[^ ^32^ ^]^ was introduced to reinforce the correlation between similar cells in each batch. Similar to the *t*‐SNE method,^[^ ^34^ ^]^ the Student *t*‐distribution kernel function with one degree of freedom was used to describe the pairwise similarity among cell points of a batch in the latent space *H*. Supposedly, cells *i* and *j* were from the same batch *B*, the similarity of cell point *h*_*j*_ to cell point *h*_*i*_ is the conditional probability *q*_*j*|*i*_, which indicates the likelihood of *h*_*i*_ choosing *h*_*j*_ as its neighbor based on their proximity in batch *B*.

(7) $$q_{j|i}=\left\{\begin{array}{cc} \frac{{\left(1+\left|\right|h_{i}-h_{j}|{|}^{2}\right)}^{-1}}{\sum_{k\in B,k\neq i}{\left(1+\left|\right|h_{i}-h_{k}|{|}^{2}\right)}^{-1}} & i\neq j\\ 0 & i=j \end{array}\right.$$

Further, the pairwise similarity is refined with an auxiliary target distribution *p*, defined as:

(8) $$p_{j|i}=\frac{q_{j|i}^{2}/\sum_{l\in B,l\neq j}q_{j|l}}{\sum_{k\in B,k\neq i}\left(q_{k|i}^{2}/\sum_{l\in B,l\neq k}q_{k|l}\right)}$$

Using Equations (7) and (8), the first KL divergence loss function is defined:

(9) $$L_{\mathrm{KL}1}=\mathrm{KL}\left(p||q\right)=\sum_{B}\sum_{i\in B}\sum_{j\in B}p_{j|i}\log\frac{p_{j|i}}{q_{j|i}}$$

As the target distribution *p* is derived from *q*, this approach adopts a self‐training strategy, which is instrumental in learning a latent space more amenable to clustering.

To enhance batch integration, a second KL divergence loss function, *L*_*KL2*_, pertaining to batch information to increase the intermixing of cells from different batches within each cluster is introduced. The proportion of cells from batch *B* in the *k*‐th soft cluster is denoted as $u_{\mathrm{Bk}}=\sum_{j\in B}p_{\mathrm{jk}}/\sum_{j\;=\;1}^{N}p_{\mathrm{jk}}$, where *p*_*jk*_ is the probability that cell *j* belongs to the *k*‐th cluster. Suppose that *v*_*B*_ is the ratio of cells in batch *B* to the total number of cells across all batches, the second KL divergence loss is then defined as

(10) $$L_{\mathrm{KL}2}=\mathrm{KL}\;\left(u||v\right)=\sum_{k}\sum_{B}u_{\mathrm{Bk}}\log\frac{u_{\mathrm{Bk}}}{v_{B}}$$

Consequently, the final objective function is defined as

(11) $$\min_{W,b,P,C}F=L_{\mathrm{NB}}+\lambda_{1}L_{A}+\lambda_{2}L_{O}+\kappa_{1}L_{\mathrm{KL}1}+\kappa_{2}L_{\mathrm{KL}2}$$

(12) $$s.t.\;\sum_{k\;=\;0}^{K}\;p_{\mathrm{jk}}=1,\;0\leq p_{\mathrm{jk}}\leq 1,\;j=1,2,\text{…},N$$

As illustrated in Figure 1, $L_{\mathrm{rec}}=\sum_{j=1}^{N}∥x_{j}-{\hat{x}}_{j}{∥}_{2}^{2}$ is initially used as the initial loss functions to pre‐train the model, establishing a latent space *H*, along with weights *W* ^(*m*)^ and biases *b* ^(*m*)^. An initial soft cell assignment matrix *P*_0_ is derived from fuzzy *k*‐means. Based on *P*_0_ and *H*, the centers *C* of *K* clusters can be calculated by using Equation (13).

(13) $$c_{k}=\frac{\sum_{j=1}^{N}p_{\mathrm{jk}}h_{j}}{\sum_{j=1}^{N}p_{\mathrm{jk}}}$$

Subsequently, *P* is updated by Equation (14) with fixed *H* and *C*.

(14) $$p_{\mathrm{jk}}=\frac{\exp\left(-\left|\right|h_{j}-c_{k}|{|}_{2}\right)}{\sum_{k=1}^{K}\exp\left(-\left|\right|h_{j}-c_{k}|{|}_{2}\right)}$$

The optimization of the final objective function F in Equation (11) utilizes back‐propagation and stochastic gradient descent (SGD) algorithms to update *W* and *b*, while maintaining *P* and *C*. Here, *W* and *b* are the weight matrix and bias of the autoencoder layers, respectively, while *P* is the targeted distribution and *C* is the centers of the fuzzy *k*‐means clustering. The input of the *m*‐th layer is updated as $x_{j}^{\left(m\right)}=W^{\left(m\right)}\;h_{j}^{\left(m-1\right)}+b^{\left(m\right)}$. Successive updates to *C* and *P* are performed with the new *W*, *b*, and *H*, iterating until *P* and *C* stabilize. The two KL divergence losses within the low‐dimensional latent space are anticipated to gradually eliminate batch effects while enhancing clustering accuracy.

### Implementation

DeepBID is implemented in Python3 using the PyTorch framework. The NB‐based autoencoder is first pretrained for 20 epochs using the Adam optimizer, with a learning rate of 10^−3^ for small datasets (<10^4^ cells) and 10^−5^ for large datasets (≥10^4^ cells). The clustering procedure is trained for another 20 epochs using the SGD optimizer at the same learning rates. DeepBID utilizes batch training techniques to train the neural network on minibatch sizes of data rather than the entire dataset at once. Here, the minibatch size for both pretraining and training is set to 128 cells similarly to scziDesk.^[^ ^45^ ^]^ To improve performance and reduce the computational cost, DeepBID selects the top 1,000 HVGs as suggested in the ZINBMM method.^[^ ^46^ ^]^ For the four hyperparameters in the final objective function, DeepBID sets *κ*_1_ =  0.01 and  *κ*_2_ =  100 for all datasets, while *λ*_1_ =  5 and *λ*_2_ =  0.01 for small datasets, *λ*_1_ =  1 and *λ*_2_ =  0.0001 for large datasets.

The encoder features two hidden fully connected layers with sizes set to 500 and 300, respectively, while the decoder mirrors the encoder structure, resulting in an autoencoder of ‘*f* − 500 − 300 − 500 − *f* ’, where *f* is the dimension of the input data. To speed up the training process and ensure the stability of results across different initializations, a pre‐training strategy is employed. Specifically, in the pre‐training stage, a ‘*f* − 500 − *f* ’ subnetwork, followed by a ‘500 − 300 − 500’ subnetwork was trained first. The pre‐trained weights *W* and biases *b* are then used as the initial parameters for the autoencoder. All experiments were conducted on a Linux workstation equipped with an Nvidia GTX 1080Ti GPU.

### Evaluation Metrics and Datasets

The clustering performance is evaluated by ARI^[^ ^34^ ^]^ and NMI,^[^ ^35^ ^]^ which are commonly used to assess the clustering of scRNA‐seq data. Given a predicted label set *V* from clustering and a true cell type label set *U* for total *n* cells, the ARI is defined as

(15) $$\begin{array}{c} \mathrm{ARI}=\frac{\sum_{i\in V,j\in U}\left(\begin{array}{c} n_{ij}\\ 2 \end{array}\right)-\left(\sum_{i\in V}\left(\begin{array}{c} a_{i}\\ 2 \end{array}\right)\sum_{j\in U}\left(\begin{array}{c} b_{j}\\ 2 \end{array}\right)\right)/\left(\begin{array}{c} n\\ 2 \end{array}\right)}{\frac{1}{2}\left(\sum_{i\in V}\left(\begin{array}{c} a_{i}\\ 2 \end{array}\right)+\sum_{j\in U}\left(\begin{array}{c} b_{j}\\ 2 \end{array}\right)\right)-\left(\sum_{i\in V}\left(\begin{array}{c} a_{i}\\ 2 \end{array}\right)\sum_{j\in U}\left(\begin{array}{c} b_{j}\\ 2 \end{array}\right)\right)/\left(\begin{array}{c} n\\ 2 \end{array}\right)} \end{array}$$

where *n*_*ij*_ is the number of cells that are present in both cluster *i* ∈ *V* and cell type *j* ∈ *U*, *a*_*i*_ is the number of cells in cluster *i*, and *b*_*j*_ is the number of cells in cell type *j*. NMI is defined as the mutual information between *U* and *V* normalized by the maximum entropy of *U* and *V*.

(16) $$\mathrm{NMI}=\frac{\sum_{i\in U,j\in V}n_{\mathrm{ij}}\log\frac{nn_{\mathrm{ij}}}{a_{i}b_{j}}}{\max\left(-\sum_{i\in U}a_{i}\log\frac{a_{i}}{n},-\sum_{j\in U}b_{j}\log\frac{b_{j}}{n}\right)}$$

To directly evaluate the effect of data integration, the Local Inverse Simpson Index (LISI) was employed,^[^ ^17^ ^]^ which featured two specific indicators: iLISI (i.e., integration LISI) and cLISI (i.e., cell‐type LISI). These indicators are used respectively to calculate the number of batches and the number of cell types within a local neighborhood. The cLISI and iLISI values are calculated for each cell, and then the distributions of the cLISI and iLISI values in all cells are obtained. The lower bound values of both iLISI and cLISI are 1. An ideal cLISI value of 1 indicates that cells of the same cell type are neighbors, whereas an iLISI of 1 indicates poor integration, suggesting that cells from the same batch are neighbors. The LISI code was downloaded from https://github.com/immunogenomics/LISI.

DeepBID's performance on five scRNA‐seq datasets that were sequenced either by a single sequencing platform or generated by multiple platforms (see Table S1, Supporting Information) was evaluated. These datasets, labeled with batch and cell type information, have been used as benchmarks for various integration or clustering tools.^[^ ^14^, ^17^, ^21^, ^22^ ^]^ Specifically, the “DC” dataset contains four types of human dendritic cells (DCs) across two batches, both sequenced by SMART‐seq2 and available from the NCBI GEO database under accession number ‘GSE94820’.^[^ ^47^ ^]^ The “cell_lines” dataset consists of three batches from two cell lines: 1) pure “Jurkat”, 2) pure “293T”, and 3) a 50/50 mix of “Jurkat” and “293T”.^[^ ^48^ ^]^ The “Sc_mixology” dataset, under GEO accession number GSE118767, includes three human lung adenocarcinoma cell lines HCC827, H1975, and H2228, mixed equally and sequenced using three different platforms (10X Chromium, CEL‐seq2 and Drop‐seq).^[^ ^49^ ^]^ The “PBMCs” dataset includes three batches of human peripheral blood mononuclear cells (PBMCs), each processed using the 10X Chromium platform but with different protocols: 3′ end v1 (3pV1), 3′ end v2 (3pV2) and 5′ end (5p) chemistries. Lastly, the “Pancreas” dataset contains five batches of human pancreatic islet cells that were collected from five independent studies using different sequencing platforms.^[^ ^50^, ^51^, ^52^, ^53^, ^54^ ^]^

As a real application, DeepBID were applied in the integrative analysis of three scRNA‐seq datasets from patients with Alzheimer's disease. The data were obtained from the NCBI GEO database under accession number GSE138852. The three scRNA‐seq datasets were derived from entorhinal cortex tissue post‐mortem and were labeled as AD1‐2 (GSM4120429, 3,028 cells), AD3‐4 (GSM4120424, 2,005 cells), and AD5‐6 (GSM4120423, 1,040 cells). Each dataset has been annotated for 8 cell types in previous studies.^[^ ^38^ ^]^ For each cell cluster, the SCANPY package with the command ‘*scanpy.tl.rank_genes_groups*(adata, method = ‘wilcoxon’)‘ was used to find DEGs (one vs others). The cell types outputted by DeepBID were manually annotated by mapping them to the authors’ clustering results.

### Method Selection and Settings for Comparison

Recent benchmarks of batch effect correction methods have shown that Harmony,^[^ ^17^ ^]^ LIGER,^[^ ^18^ ^]^ Seurat4,^[^ ^10^ ^]^ and scVI^[^ ^20^ ^]^ outperform others in scRNA‐seq data integration.^[^ ^5^, ^7^ ^]^ Additionally, DESC^[^ ^22^ ^]^ is a newly designed method for deep clustering‐based integration. Therefore, these five methods were selected for comparison with DeepBID. Default parameters were used unless otherwise specified. For methods with alternative parameter settings recommended in their respective publications for specific datasets, those recommendations were adhered to. For example, although the default value of the *τ* hyperparameter in Harmony is 0, it was set to 5 for pancreas analysis. The number of iNMF factors for LIGER, the number of integration anchors for Seurat4, and the number of principal components (PCs) for Harmony were all fixed at 30. For data integration methods without inherent clustering algorithms, the Leiden algorithm^[^ ^55^ ^]^ from SCANPY (using the “*scanpy.tl.leiden*” function) for scVI and the clustering methods of Seurat4 (using the “*FindNeighbors*” and “*FindClusters*” functions) for Seurat4, Harmony, and LIGER to cluster the integrated data using default parameter settings were employed. For DESC, its built‐in clustering strategy was utilized with default settings. The code used for the five methods is provided in Table S6 (Supporting Information).

## Conflict of Interest

The authors declare no conflict of interest.

## Author Contributions

S.Z. and Y.C. performed conceptualization; S.Z. performed methodology; S.Z. and Q.L. acquired software; S.Z., Q.L., G.Z., and Y.C. performed the formal analysis; Y.C. and S.Z. performed writing‐original draft preparation; Y.C. and S.Z. wrote‐review and editing; All authors have read and agreed to the published manuscript.

## Supporting information

Supporting Information

Supplemental Table 1

## Data Availability

Data used in this study are available in Gene Expression Omnibus (GEO) with the accession numbers GSE94820, GSE81076, GSE85241, GSE86469, GSE84133, GES138852 and on the 10x genomics official websites at https://www.10xgenomics.com/resources/datasets (access on June 2023). The code of DeepBID is available at https://github.com/shaoqiangzhang/DeepBID.
