# Supplementary material for: Deep Batch Integration and Denoise of Single‐Cell RNA‐Seq Data
Source: Adv Sci (Weinh). 2024 May 22;11(29):2308934. doi: 10.1002/advs.202308934 (PMC11304254; doi:10.1002/advs.202308934)
Supplement: Supplementary file 1 — Supporting Information [file ADVS-11-2308934-s002.pdf]

ADVANCED SCIENCE
Open Access

Supporting Information

for *Adv. Sci.*, DOI 10.1002/advs.202308934

Deep Batch Integration and Denoise of Single-Cell RNA-Seq Data

Lu Qin, Guangya Zhang, Shaoqiang Zhang and Yong Chen**

SUPPLEMENTARY MATERIALS

Deep Batch Integration and Denoise of Single-Cell RNA-seq Data

Lu Qin[1], Guangya Zhang[1], Shaoqiang Zhang[1]*, Yong Chen[2]*

[1]College of Computer and Information Engineering, Tianjin Normal University, Tianjin 300387, China
[2] Department of Biological and Biomedical Sciences, Rowan University, NJ 08028, USA

* To whom correspondence should be addressed:
S. Zhang (zhangshaoqiang@tjnu.edu.cn); Y. Chen (chenyong@rowan.edu)

<u>This supplementary file includes Table S1-S6, Figure S1-S5.</u>

Table S1. Detailed information of scRNA-seq datasets used.

Table S2. The median iLISI scores of each dataset integrated by each method.

Table S3. The median cLISI scores of each dataset integrated by each method.

Table S4. The ARI and NMI scores of each dataset after batch integration and clustering.

Table S5. Running time comparison of six methods across five datasets.

Table S6. Core code used by five methods for batch integration and clustering.

Figure S1. NMI scores for seven different κ_1 settings across five datasets with fixed values of λ_1, λ_2 and κ_2.

Figure S2. NMI scores for seven different κ_2 settings across five datasets with fixed values of λ_1, λ_2 and κ_1.

Figure S3. UMAP plots on the “DC” dataset that has 4 cell types.

Figure S4. UMAP plots on the Pancreas” dataset that has 17 cell types.

Figure S5. UMAP plots on the “PBMCs” dataset that has 15 cell types.

Supplementary Table S1. Detailed information of used scRNA-seq datasets. CT: Cell Types.

Name	Data Source	Platforms	URL	# Cell	# CT
DC	Human dendritic cells	SMART-seq2	https://www.ncbi.nlm.nih.gov/geo/query/acc.cgi?acc=GSE94820	286	4
		SMART-seq2		283	
Cell_lines	Jurkat	10X Genomics	https://support.10xgenomics.com/single-cell-gene-expression/datasets/1.1.0/jurkat	3258	2
	HEK293T	10X Genomics	https://support.10xgenomics.com/single-cell-gene-expression/datasets/1.1.0/293t	2885	
	50% Jurkat + 50% HEK293T	10X Genomics	https://support.10xgenomics.com/single-cell-gene-expression/datasets/1.1.0/jurkat:293t_50:50	3388	
Sc_mixology	Mixture of HCC827, H1975 and H2228	10X Genomics	https://github.com/LuyiTian/sc_mixology	902	3
		CEL-seq2		274	
		Drop-seq		225	
PBMCs	3pV1	10X Genomics (3')	https://support.10xgenomics.com/single-cell-gene-expression/datasets/1.1.0/pbmc6k	5419	15
	3pV2	10X Genomics (3')	https://support.10xgenomics.com/single-cell-gene-expression/datasets/2.1.0/pbmc8k	8381	
	5p	10X Genomics (5')	https://support.10xgenomics.com/single-cell-vdj/datasets/2.2.0/vdj_v1_hs_pbmc_5gex	7726	
Pancreas	Human pancreatic islet cells	CEL-seq	https://www.ncbi.nlm.nih.gov/geo/query/acc.cgi?acc=GSE81076	946	11
		CEL-seq2	https://www.ncbi.nlm.nih.gov/geo/query/acc.cgi?acc=GSE85241	2238	
		SMART-seq2	https://www.ebi.ac.uk/gxa/sc/experiments/E-MTAB-5061/	2114	
		Fluidigm C1	https://www.ncbi.nlm.nih.gov/geo/query/acc.cgi?acc=GSE86469	513	
		inDrops	https://www.ncbi.nlm.nih.gov/geo/query/acc.cgi?acc=GSE84133	8564	

Supplementary Table S2. The median iLISI scores of each dataset after batch integration.

Method\Dataset	DC	Cell_lines	Sc_mixology	Pancreas	PBMCs
DeepBID	**1.379**	**1.429**	1.000	**2.475**	**2.728**
Harmony	1.163	1.000	1.000	1.000	1.178
Seurat	1.175	1.004	1.000	1.020	1.012
LIGER	1.071	1.370	**1.273**	1.011	1.073
scVI	1.302	1.259	1.012	1.000	1.029
DESC	1.016	1.000	1.000	1.000	1.857

Supplementary Table S3. The median cLISI scores of each dataset after batch integration.

Method\Dataset	DC	Cell_lines	Sc_mixology	Pancreas	PBMCs
DeepBID	**1.845**	3.098	**2.466**	**2.862**	4.728
Harmony	2.055	1.947	3.045	3.014	4.153
Seurat	2.016	**1.649**	3.977	2.885	3.593
LIGER	1.973	7.684	6.794	2.885	7.022
scVI	1.973	4.691	4.609	3.323	6.590
DESC	1.982	2.923	4.553	3.098	**3.098**

Supplementary Table S4. The ARI and NMI scores of each dataset after batch integration and clustering.

Datasets	Indices	Harmony	Seurat	LIGER	scVI	DESC	DeepBID
DC	NMI	0.65246	0.73514	0.84188	0.65721	0.80453	**0.84437**
	ARI	0.78790	0.84787	0.63021	0.78302	0.87448	**0.89067**

Sc_mixology	NMI	0.48738	0.60919	0.56768	0.55534	0.74793	**0.98498**
	ARI	0.44375	0.61212	0.29195	0.38914	0.27613	**0.99146**
Cell_lines	NMI	0.58549	0.36147	0.37397	0.22004	0.51159	**0.97854**
	ARI	0.44557	0.31408	0.07088	0.13607	0.36330	**0.99821**
PBMCs	NMI	0.71250	0.57896	0.60484	0.69486	0.68155	**0.72594**
	ARI	0.63037	0.42125	0.43478	0.70293	0.48305	**0.73191**
Pancreas	NMI	0.65348	0.68489	0.45291	0.69524	0.70117	**0.71923**
	ARI	0.36379	0.46508	0.60950	0.50869	0.23283	**0.62265**

Supplementary Table S5. Running time comparison of six methods across five datasets. The cell numbers for each dataset are provided in parentheses, and the running time is measured in seconds (s). The running time of each tool was recorded for their core code processing, excluding data preprocessing time.

Name	DC (569)	Sc_mixology (1401)	Cell_lines (9531)	Pancreas (14375)	PBMCs (21526)
DeepBID	4.45s	7.85s	140.94s	302.3s	416.5s
Harmony	1.76s	2.4s	40.9s	85.1s	93.7s
Seurat	1.09s	1.62s	21.59s	24.87s	40.83s
LIGER	59.51s	74s	210s	417.51s	482.86s
DESC	31.4s	31.9s	326.5s	574.3s	586.1s
scVI	14.9s	38.9s	326.2s	667.3s	883.4s

Supplementary Table S6. Core code used by five methods for batch integration and clustering.

Method	Core code
LIGER	scRNA_liger <- merge(batches)<br>scRNA _liger <- NormalizeData(scRNA _liger)<br>scRNA _liger <- FindVariableFeatures(scRNA _liger)<br>scRNA _liger <- ScaleData(scRNA _liger, split.by = "orig.ident", do.center = F)<br>scRNA _liger <- RunOptimizeALS(scRNA _liger, k = 30, lambda = 5, split.by = "orig.ident")<br>scRNA _liger <- RunQuantileNorm(scRNA _liger, split.by = "orig.ident")<br>scRNA_liger <- FindNeighbors(scRNA_liger,reduction = "iNMF",k.param = 10,dims = 1:30)<br>scRNA_liger <- FindClusters(scRNA_liger)
Seurat	scRNA.anchors <- FindIntegrationAnchors(object.list = scRNAlist)<br>scRNA_seurat <- IntegrateData(anchorset = scRNA.anchors)<br>scRNA_seurat <- RunUMAP(scRNA_seurat, dims = 1:30)<br>scRNA_seurat <- FindNeighbors(scRNA_seurat, dims = 1:30) %>% FindClusters()
Harmony	scRNA_harmony <- merge(batches)<br>scRNA_harmony <- NormalizeData(scRNA_harmony) %>% FindVariableFeatures() %>% ScaleData() %>% RunPCA(verbose=FALSE)<br>scRNA_harmony <- RunHarmony(scRNA_harmony, group.by.vars = "orig.ident")<br>scRNA_harmony <- RunUMAP(scRNA_harmony, reduction = "harmony", dims = 1:30)<br>scRNA_harmony <- FindNeighbors(scRNA_harmony, reduction = "harmony", dims = 1:30) %>% FindClusters()
DESC	scanpy.pp.scale(adata,max_value=6) # require scanpy<br>save_dir=" "<br>adata=DESC.train(adata, dims=[adata.shape[1],64,32], tol=0.005, n_neighbors=10, batch_size=256, louvain_resolution=[0.8,1.0], save_dir=str(save_dir), do_tsne=True, learning_rate=200, use_GPU=True, num_Cores=1, num_Cores_tsne=4, save_encoder_weights=False, save_encoder_step=3, use_ae_weights=False, do_umap=False)
scVI	scanpy.pp.highly_variable_genes(adata, flavor="seurat_v3", n_top_genes=2000, layer="norm_data", batch_key="batch", subset=True,)<br>scvi.model.SCVI.setup_anndata(adata,batch_key="batch")<br>vae = scvi.model.SCVI(adata, n_layers=2, n_latent=30, gene_likelihood="nb")<br>vae.train()

Supplementary Figure S1. NMI scores for seven different κ_1 settings across five datasets with fixed values of λ_1, λ_2 and κ_2.

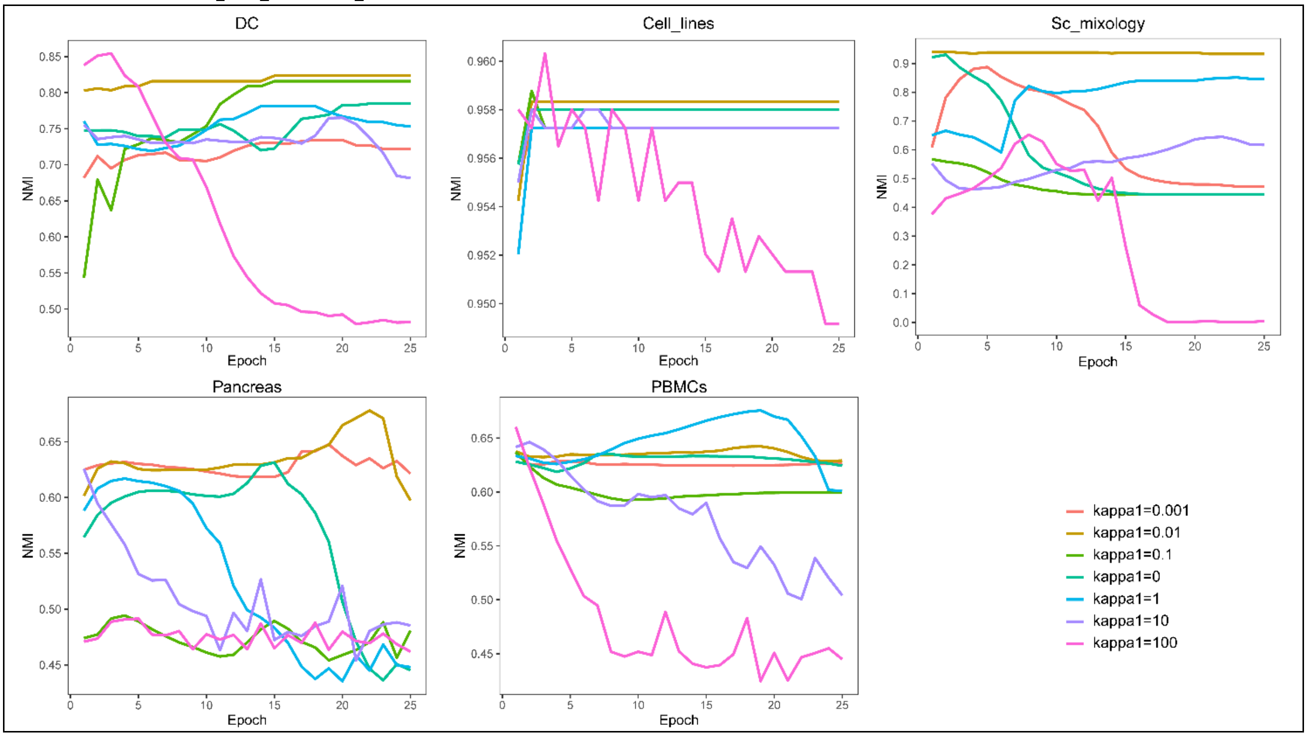

Supplementary Figure S2. NMI scores for seven different κ_2 settings across five datasets with fixed values of λ_1, λ_2 and κ_1.

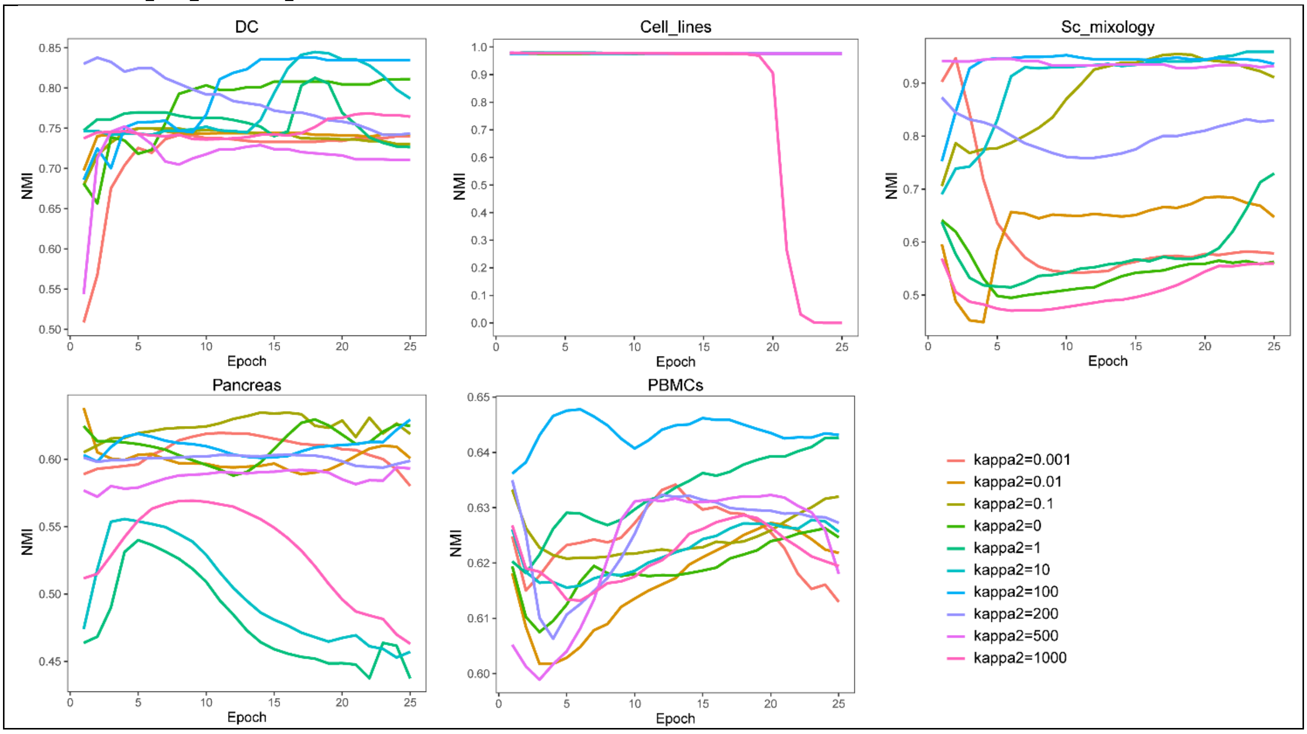

Supplementary Figure S3. UMAP plots on the "DC" dataset that has 4 cell types. The first column illustrates the original cell type labels by method, the second column displays the labels for 2 batches, and the third column shows the clustering outcomes after batch effect removal.

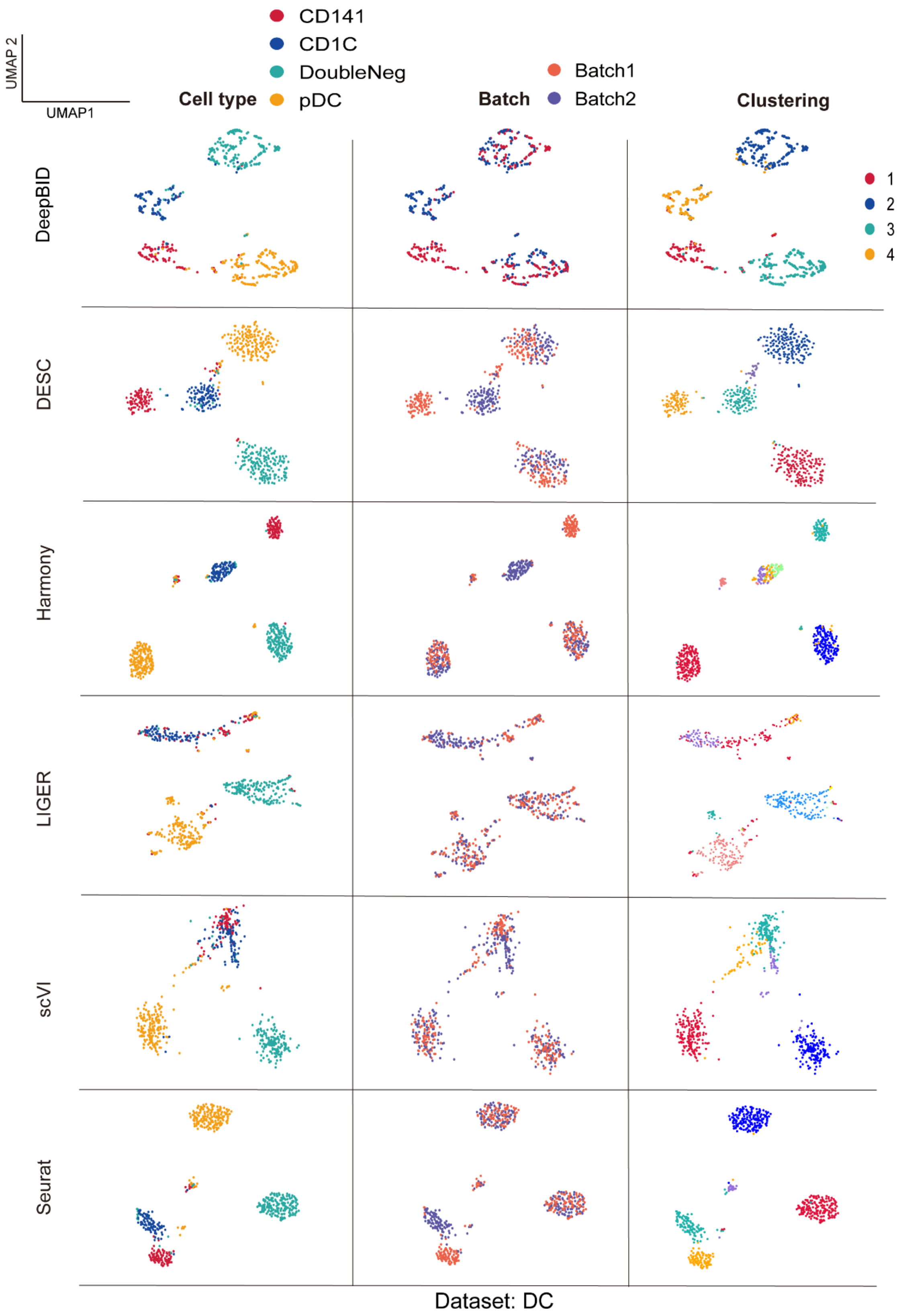

Supplementary Figure S4. UMAP plots on the "Pancreas" dataset that has 17 cell types. The first column illustrates the original cell type labels for each method, the second column displays the labels for 5 batches, and the third column shows the clustering outcomes after batch effect removal.

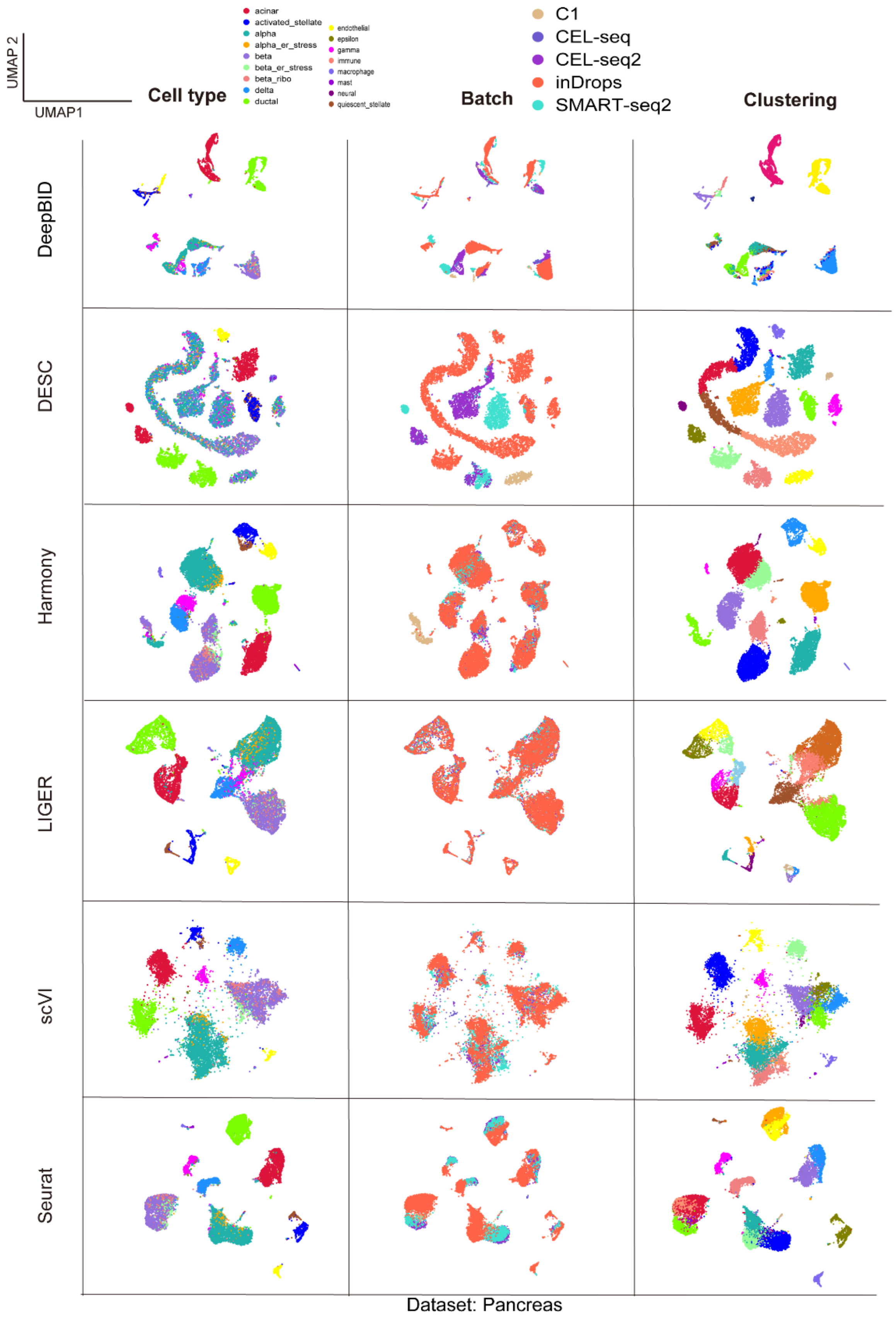

Supplementary Figure S5. UMAP plots on the "PBMCs" dataset that has 15 cell types. The first column illustrates the original cell type labels for each method, the second column displays the labels for 3 batches, and the third column shows the clustering outcomes after batch effect removal.

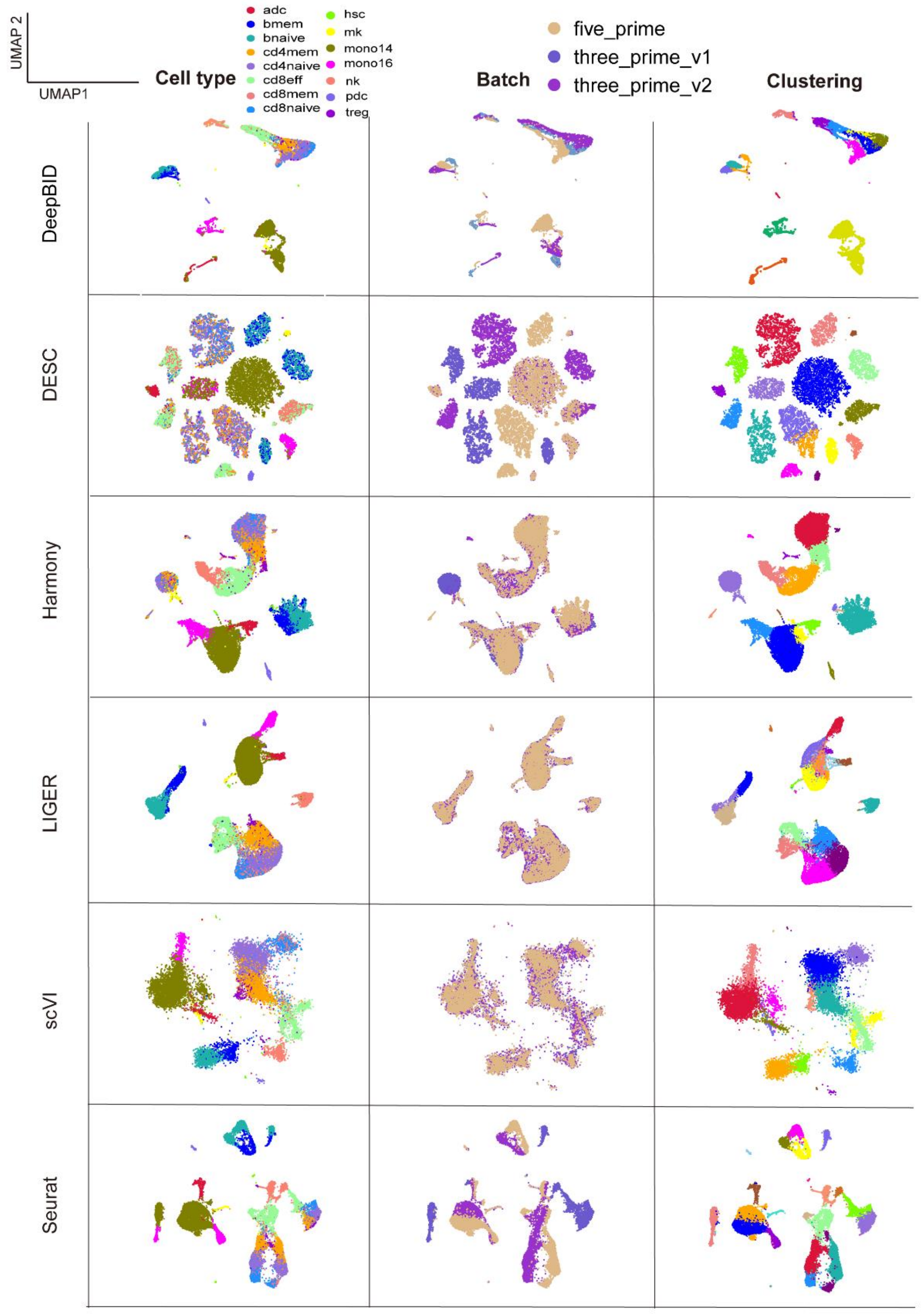

Dataset: PBMCs
